# How to Translate Basic Knowledge into Clinical Application of Biologic Therapy in Spondyloarthritis

**DOI:** 10.1155/2013/369202

**Published:** 2013-06-11

**Authors:** Chung-Tei Chou

**Affiliations:** Division of Allergy-Immunology-Rheumatology, Department of Medicine, Veterans General Hospital, No. 201, Section 2, Shipai Road, Beitou District, Taipei 112, Taiwan

## Abstract

Spondyloarthritis (SpA) is a family of many diseases, and these diseases share some clinical, genetic, and radiologic features. The disease process in the spine at the beginning is spinal inflammation, in which TNF**α** is the principal cytokine involved. Therefore, the dramatic clinical and pathologic response of anti-TNF**α** therapy in SpA is based upon the presence of increased TNF**α** in synovial tissues and sacroiliac joints, which perpetuates chronic inflammation. The increased Toll-like receptors (TCR) 2 and 4 in the serum, peripheral blood mononuclear cells, or synovial tissues of ankylosing spondyloarthritis (AS) or SpA patients suggest that SpA is highly associated with innate immunity. Any drug including anti-TNF**α** blocker which can downregulate the TCR, infiltrated neutrophils, or CD163+ macrophages in the synovial tissue is the rationale for the management of SpA. Like rheumatoid arthritis, the increased TH22 and TH17 cells either in blood, synovial fluid, or synovial tissues were also demonstrated in SpA. Thus, TH17 and TH22 may be reasonable cellular targets for therapeutic intervention. Drugs (anti-IL6R or anti-IL6) which can reduce the binding of IL6 and IL6R to the cell surface may be beneficial in SpA. Many proteins are implicated in the new bone formation (syndesmophyte) or ankylosis in AS or SpA. The enhanced BMP and Wnt pathway will activate osteoblasts which promote the new bone formation. However, no drug including anti-TNF**α** can stop or prevent the syndesmophyte in AS. In summary, looking for new targeting therapies for either anti-inflammation (beyond anti-TNF) or anti-bone formation (including anti-TGF**β** or PDGF) is warranted in the future.

## 1. Introduction

Spondyloarthritis (SpA) is a family of many diseases that includes ankylosing spondylitis (AS), reactive arthritis (ReA), psoriatic arthritis (PsA), inflammatory bowel disease (IBD), and undifferentiated arthritis (USpA) [1–3]. These diseases share some clinical, genetic, and radiologic features. The most common and important prototype in SpA is AS. Defining preaxial SpA by using the new AS criteria has recently become an important issue in the early diagnosis and management of SpA.

The pathologic process of AS or SpA can be divided into 3 stages: stage 1 is spinal inflammation, in which TNF*α* is the principle cytokine involved [4]. Stage 2 is erosion, in which cathepsin K or matrix metalloproteinase (MMP) may contribute. Stage 3 is abnormal bone remodeling, which can exhibit new bone formation (syndesmorphytes). Bone morphogenic protein (BMP) and Wnt protein are 2 major proteins that may enhance osteoblast activity with new bone formation.

To reduce inflammation and pain, nonsteroidal anti-inflammatory drugs (NSAIDs) are the first-line remedy in AS or SpA [3, 5–7]. However, only 70%–80% of patients have a response. The synthetic disease modifying antirheumatic drugs (DMARDS), including methotrexate, salazopyrin, and leflunomide, are active therapy for PsA or AS with peripheral arthritis but no efficacy for axial SpA [8–10].

In the past 10 years, TNF*α* inhibitors have been demonstrated to be very effective in rheumatoid arthritis (RA), AS, and PsA [3, 7, 11–13]. Five anti-TNF*α* blockers have been approved and are used in the management of inflammatory arthritis. The TNF*α* blocker is superior in pain relief, joint function, and life quality improvement and in reducing ESR and CRP and decreasing inflammation as seen on MRI, compared to conventional therapy. Moreover, the rapid onset (usually 2 weeks after injection) and persistence of drug survival are two other major benefits.

From NSAIDs and DMARDs to the recent biologic therapy, what is the mechanism of these drugs that may lead to success or failure in the management of patients with RA or SpA? Why would the drugs be effective in the early stage but then lose efficacy at a later stage? In this review paper, the translation of basic knowledge to the clinical application of biologic therapy may provide some answers.

## 2. The Role of TNF*α* in SpA and Why Anti-TNF*α* Is Very Useful in SpA

Accumulating evidence has shown that TNF*α* plays a pivotal role in inflammatory arthritis [14, 15]. Many cells from the inflammatory synovium, when activated, can release different cytokines. Among them, TNF*α* is a potent proinflammatory cytokine exerting pleiotropic effects on various cell types. Our previous study on hip synovitis in AS and knee arthritis in RA and osteoarthritis (OA) demonstrated that TNF*α* expression was prominent in synovial lining cells in both RA and AS [16]. The MMP3 and CD68+ cells were significantly increased in AS compared to OA. A short-term, open-label and multiple center study in Taiwan demonstrated that etanercept was very effective in Chinese patients with AS [17]. In addition, laboratory investigation also confirmed that etanercept could significantly decrease the serum levels of IL6 and MMP3 [18]. German investigators reported that TNF*α* was overexpressed in synovial tissues that were obtained from the sacroiliac joint of AS patients through CT-guided needle biopsy [15]. Since 2000, many clinical trials have demonstrated that anti-TNF*α* therapy could significantly reduce spine and joint inflammations [3, 13, 19, 20]. This clinical response paralleled the pathologic changes [21–23]. Early research with 8 SpA patients found that after infliximab therapy, the reduction of lining cell hyperplasia, vascularity, and mononuclear cells infiltration was very dramatic and that that had a good correlation with the clinical response [23]. A recent study evaluating the effect of adalimumab treatment on synovial tissues suggested that CD3 T cells and MMP-13 might be used as biomarkers that are sensitive to changes after treatment [24]. The abundant synovial expression of RANKL and OPG in SpA was also dramatically decreased after the patients had received an anti-TNF*α* drug [25].

Therefore, the implication of the efficacy of anti-TNF*α* therapy in SpA is based on the presence of increased TNF*α* in synovial tissues and sacroiliac joints, which perpetuates chronic inflammation. The dramatic clinical response and long-term drug survival of TNF*α* blockers in those active AS patients confirmed that anti-TNF*α* therapy is the best treatment in AS or other SpA diseases [13, 26].

## 3. The Role of Innate Immunity in SpA and Future Targeting Therapy

Toll-like receptor (TLR) is a type I transmembrane receptor and can recognize the pathogen-associated molecular pattern or danger-associated molecular pattern [27–29]. Several TLRs have been identified. Among them, TLR4 most commonly binds with the ligand, lipopolysaccharide (LPS) of Gram-negative bacteria. TLR can protect the host against bacterial infection, but when inappropriately secreted, it can cause chronic inflammation and autoimmunity. The engagement of TLRs results in activation of the NF-k*β* pathway, which promotes secretion of proinflammatory cytokines, such as IL6, IL12, TNF, and IFn-r, that may drive inflammation in AS. TNF*α* may increase TLR expression.

In one animal study, the enhanced B-cell TLR7 expression permits the specific development of Abs to RNA/protein complexes [30]. Zhao et al. studied TLR2 and TLR4 expression and TH1/TH2-related cytokines in bronchoalveolar lavage fluid (BALF) and peripheral blood and concluded that TLR2 and TLR4 are involved in acute inflammatory response in the lung histology in mice [31]. Study on murine salmonella intestinal infection by Shi et al. suggested in contrast to TLR5 as a “carrier of salmonella,” TLR 11 works as a “blocker of salmonella” to prevent highly invasive salmonella from penetrating into the murine Peyer's patches [32]. 

Several studies have shown that TLR2 or TLR4 was significantly increased in the serum, peripheral blood mononuclear cells, or synovial tissues of AS and SpA patients [33, 34]. When those patients were treated with anti-TNF*α* blockers, significantly reduced TLR2, TLR4, or TLR5 was seen [33]. Innate immunity has been suggested to play a pivotal role in AS. This is based on the evidence of early synovial infiltration of neutrophils, CD163+ macrophages, and CD17+ mast cells, which are innate immune cells [35–38]. Those cells are not only present in synovial tissue, but they are already primed and activated and cause inflammation. The rationale for the management of inflammatory arthritis, particularly at the early stage of SpA [39], includes decreasing chemokine or chemokine receptors in activated innate-related immune cells, increasing the apoptosis of infiltrated macrophages (CD163+), and downregulating TLR expression.

Whether the targeting therapy for TLR can modify the SpA clinical features remains unknown, and this may need further study to elucidate.

## 4. Are IL17, IL23, and TH17 Cells, and Recently TH22 Cells, Playing an Important Role in AS or SpA, and How Will Future Cellular Targeting Therapy Be?

TH1 cells are important in the pathogenesis of RA. Another subset of CD4+ T cells, CD17 cells, was recently implicated to be associated with the inflammation in RA [40–42]. The proinflammatory cytokine IL17, which is released from TH17 cells, can stimulate many cells, including synovial fibroblasts, macrophages, and synovial lining cells, to produce proinflammatory cytokines (TNF*α*, IL6, etc.) and RANKL, as well as GMCSF, which can enhance osteoclast numbers and activity. Several studies have explored the relationship between IL17 and IL23 and SpA [43–48]. In AS or PsA, increased serum IL17 and TH17 cells were demonstrated [43, 46]. Increased IL23R was found in psoriasis skin, and anti-IL23R could improve the psoriasis [49]. Chinese investigators and our group as well have shown that serum IL17 and IL23 levels were significantly increased in AS [44, 47, 50]. Using RT-PCR, they demonstrated that IL23R P19mRNA was significantly increased in the PBMC of AS patients. Therefore, IL17, TH17 cells, and IL23 are important in the pathogenesis of AS. This has been translated into the development of a new targeting therapy, secukinumab, which has shown efficacy in early clinical trial [51].

In addition to TH1 and TH17 cells, the TH22 cells, a new human T-helper subset, were recently defined [52–55]. The naive T cell in the presence of IL6 and TNF*α* can differentiate toward TH22 cells. Increased IL22 from activated TH22 cells was demonstrated in the blood of RA patients and in the skin of psoriasis patients [52, 55]. The results of an investigation of TH22 in ethnic Chinese with AS showed that compared to OA, the blood TH17 and TH22 cells and serum IL22 were all significantly elevated [55]. In addition, a positive correlation between TH22 cells and TH17 cells or TH17 cells and IL22 was disclosed. Therefore, we suggest that TH22 cells, TH17 cells, and their products, IL17 as well as IL22, are implicated in the pathogenesis of AS. TH22 and TH17 cells may be reasonable cellular targets for therapeutic intervention. However, this requires further elucidation through future clinical trials.

Since TH17 cells are belonging to the upstream inflammatory cells and their products, IL17 can affect the downstream proinflammatory cytokines (TNF*α*, IL1, IL6, etc.) secretion, and targeting therapy for IL17 is the rationale to suppress the inflammation in AS or SpA [56].

## 5. Beyond TNF*α*, the Role of IL6 in AS and IL6R-Targeting Therapy (Tocilizumab) in AS

A previous study showed that the disease-activity biomarkers in AS were ESR and CRP. A recent review paper indicated that CRP, IL6, and VEGF were inflammation-related markers. Among them, CRP is the most important because CRP levels in serum can predict persistent inflammation, subsequent syndesmophyte formation, and treatment response to anti-TNF*α* drugs in AS patients [18, 57–59]. CRP is mainly produced by hepatocytes, and 2 cytokines, including IL1 and IL6, are major stimulators. Early studies have shown that serum IL6 was increased in AS. We did a small cohort study treating ethnic Chinese AS patients with etanercept. The serum IL6 and MMP3 were significantly increased in the AS patients compared to the control, and after 12 weeks of treatment with etanercept, a significant decrease in IL6 and MMP3 was demonstrated [18]. Tocilizumab (anti-IL6R) has already been proven as a potential therapy in patients with RA [60]. The anti-IL6R drug can exert its biological effect by reducing the binding of IL6 and IL6R to the cell surface, which is the process of initiating the signal transduction for cell activation. Anti-IL6R was beneficial in AS patients but only reported in small case studies, and this awaits further large randomized control trial for confirmation [61–63].

## 6. Abnormal Bone Remodeling in AS and Can We Prevent Aberrant New Bone Formation in AS?

Unlike RA, abnormal bone remodeling can occur in AS [14, 64, 65]. In RA, the increased TNF*α* can increase DKK1, which is the natural inhibitor of Wnt protein. When the Wnt *β*-catenin signal increases, it will activate osteoblasts, which cause new bone formation. In RA, the high signal of RANKL and low signal of Wnt (due to the increased DKK1) persistently stimulate osteoclast activity, but have a low probability of stimulating osteoblasts. In AS, the lower DKK1, which enhances Wnt protein, and the high signal of BMP strongly promote osteoblasts and then the development of new bone formation. Recent review paper suggested that profibrotic mediators, including transforming growth factor (TGF*β*), BMP, and platelet-derived growth factor (PDGF) may induce the myofibroblast phenotype and drive new bone formation [66].

The GESPIC study, with a 2-year followup of AS patients in Germany, demonstrated that with lower DKK1 there is more opportunity to develop syndesmophytes [67]. A similar study from Korea also found that DKK1 was lower in AS patients than in the control [68]. Sclerostin, another molecule like Dkk1, can suppress the Wnt pathway. Appel measured sclerostin in osteocytes and serum in RA, AS, and OA patients and again found that sclerostin was significantly lower in AS patients than in RA and OA patients and healthy controls [69]. The one molecule that can enhance osteoblasts is BMP. Our previous study demonstrated that serum BMP levels were significantly increased in AS patients, particularly in patients with spinal fusion or bamboo spine [58, 70]. However, until now, no targeting therapy for BMP, TGFB, and PDGF is available for the clinical trial to see whether they can suppress the syndesmophyte.

**Figure 1:**
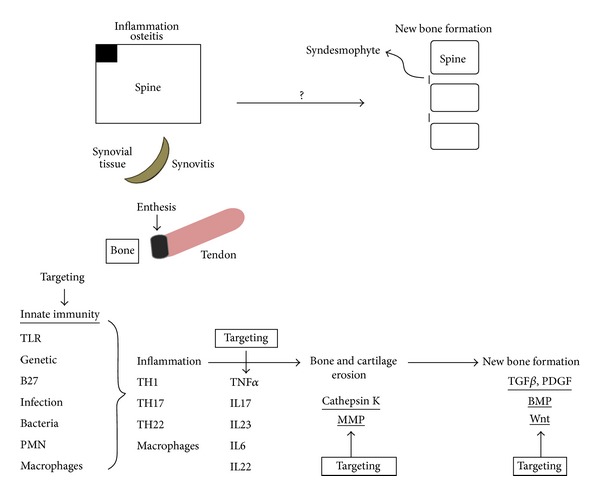
SpA: immunopathogenesis and the potential sites for targeting therapy.

The immunopathogenesis and potential sites for targeting therapy is shown in Figure 1. As of the present, no drugs or biologic therapies are available to prevent or stop the new bone formation in AS. The 3 anti-TNF*α* blockers have been used in AS, but no one has proved to be effective in suppressing syndesmophytes [71]. Looking for new targeting therapies for those bone-formation proteins may hopefully be able to stop the excessive bone remodeling in AS.
